# RGB-image method enables indirect selection for leaf spot resistance and yield estimation in a groundnut breeding program in Western Africa

**DOI:** 10.3389/fpls.2022.957061

**Published:** 2022-08-04

**Authors:** Emmanuel Kofi Sie, Richard Oteng-Frimpong, Yussif Baba Kassim, Doris Kanvenaa Puozaa, Joseph Adjebeng-Danquah, Abdul Rasheed Masawudu, Kwadwo Ofori, Agyemang Danquah, Alexandre Brice Cazenave, David Hoisington, James Rhoads, Maria Balota

**Affiliations:** ^1^Council for Scientific and Industrial Research-Savanna Agricultural Research Institute, Nyankpala, Ghana; ^2^West Africa Centre for Crop Improvement (WACCI), University of Ghana, Legon, Ghana; ^3^Bayer Crop Science, Stanton, MN, United States; ^4^Feed the Future Innovation Lab for Peanut, University of Georgia, Athens, GA, United States; ^5^School of Plant and Environmental Sciences, Virginia Tech, Tidewater Agricultural Research and Extension Center, Suffolk, VA, United States

**Keywords:** RGB image, phenotyping, leaf spot, diseases, groundnut, selection

## Abstract

Early Leaf Spot (ELS) caused by the fungus Passalora arachidicola and Late Leaf Spot (LLS) also caused by the fungus Nothopassalora personata, are the two major groundnut (*Arachis hypogaea* L.) destructive diseases in Ghana. Accurate phenotyping and genotyping to develop groundnut genotypes resistant to Leaf Spot Diseases (LSD) and to increase groundnut production is critically important in Western Africa. Two experiments were conducted at the Council for Scientific and Industrial Research-Savanna Agricultural Research Institute located in Nyankpala, Ghana to explore the effectiveness of using RGB-image method as a high-throughput phenotyping tool to assess groundnut LSD and to estimate yield components. Replicated plots arranged in a rectangular alpha lattice design were conducted during the 2020 growing season using a set of 60 genotypes as the training population and 192 genotypes for validation. Indirect selection models were developed using Red-Green-Blue (RGB) color space indices. Data was collected on conventional LSD ratings, RGB imaging, pod weight per plant and number of pods per plant. Data was analyzed using a mixed linear model with R statistical software version 4.0.2. The results showed differences among the genotypes for the traits evaluated. The RGB-image method traits exhibited comparable or better broad sense heritability to the conventionally measured traits. Significant correlation existed between the RGB-image method traits and the conventionally measured traits. Genotypes 73–33, Gha-GAF 1723, Zam-ICGV-SM 07599, and Oug-ICGV 90099 were among the most resistant genotypes to ELS and LLS, and they represent suitable sources of resistance to LSD for the groundnut breeding programs in Western Africa.

## Introduction

Groundnut is a nutritious crop with high protein (12% to 36%) and oil (36% to 54%) content and an important food crop worldwide. Groundnut seed also plays a crucial role in providing, vitamins, minerals, unsaturated oil and plant protein for many people in Ghana (Gaikpa et al., 2021). The nutritional value of groundnut renders it an essential component in the diet of rural people in Northern Ghana, as it complements the protein intake requirement in their mostly cereal-based diet. Daily consumption of groundnut contributes immensely to reducing protein deficiency and malnutrition in this country. In addition to increasing food and nutritional security, groundnut plays a pivotal role in the life of small-holder farmers in Ghana as a suitable vehicle for making improvements in the areas of poverty alleviation (Tyroler, 2018). In spite of its numerous benefits, cultivation and productivity of groundnut in Ghana and the world is largely hindered by numerous biotic factors (Oppong-Sekyere et al., 2015). Early Leaf Spot (ELS) caused by the fungus Passalora arachidicola previously known as Cercospora arachidicola and Late Leaf Spot (LLS) also caused by the fungus Nathopassalora personata, known previously as Cercosporidium peronatum (Denwar et al., 2021) diseases represent major destructive groundnut diseases. For example, LLS can cause loss in yield between 30 to 70% for susceptible varieties under disease conducive environmental conditions (Mugisha et al., 2004; Singh et al., 2011). The challenge to feed the growing human population in the face of numerous factors that limit the quality and quantity of groundnut production such as ELS and LLS diseases is an uphill task. Both ELS and LLS reduce the available leaf area for photosynthesis and therefore leads to defoliation and yield loss. Groundnut breeders are making efforts to screen large numbers of accessions for the development of ELS and LLS resistant varieties. Conventionally, ELS and LLS assessment in breeding programs includes visual scoring of disease severity. Nonetheless, this approach is error-prone, i.e., it depends on the evaluator experience and ability to capture small genotypic differences, it is time-consuming, and may not be able to capture adequately the physiological status of the plant (Araus, 2018). Repeatedly, the conventional methods for LSD screening have been reported as difficult to capture genotypic differences due to the partial and polygenic nature of these diseases (Dwivedi et al., 2002). Because of this, they may reduce rather than improve the efficacy of the marker-assisted selection. Unfortunately, many of the plant breeding programs in developing countries mostly rely on only conventionally recorded phenotypic data before transcribing the data into usable forms (Rife and Poland, 2015). Such data collection methods are expensive and laborious (Araus, 2018; Awada et al., 2018). Less experience evaluator will take a longer time to arrive at ELS and LLS as compare to imaging. Moreover, even if it is an experience person doing the scoring, because of the subjective nature of visual scoring, it is difficult to give the same score to the same plot scored at different time points either by the same rater or a different rater. Red-Green-Blue (RGB)-image technique therefore offers the chance to standardized ELS and LLS measurements and provides a better way to objectively quantify leaf spot severity than the visual method.

Similarly, in Ghana and other African countries, the breeding programs are in critical need for innovative techniques to improve yield and quality of groundnut. Application of RGB-image method, i.e., the science of making measurements from photographs, for automatic phenotyping may overcome the flaws of the current conventional phenotyping methods. RGB-image method saves significant time, decreases the cost of data collection, and offers the benefits of non-destructive measurements, regular assessment, accurate observations and direct storage of data (Araus, 2018). RGB-image method has been successfully used as a powerful evaluation tool for screening drought tolerance and yield in winter wheat in Texas (Balota et al., 2007), groundnut in Virginia, United States (Balota and Oakes, 2017), and groundnut LSD in Egypt (Omran, 2017). However, the effectiveness of RGB-image method for groundnut ELS and LLS selection in Ghana is yet to be exploited. The objective of this study was to explore the effectiveness of using RGB-image method as a high-throughput phenotyping tool for the assessment of groundnut LSD and yield in a breeding program in Ghana.

## Materials and methods

### Location of experiments, groundnut genotypes, and experimental design

Two experiments were conducted between June 2020 and October 2020 at Nyankpala, located in the Tolon district of Northern region of Ghana. Nyankpala is located at 09° 25′ 41″ N, 00° 58′ 42″ W, and altitude of 183 m above the sea level. The soils of the experimental site belong to Ferric Luvisols of the Tingoli series with a brown color, moderately drained, and free from concretions (Atakora and Kwakye, 2016). The Northern region of Ghana is characterized by a relatively dry climate with unimodal rainfall ranging between 900 and 1,200 mm annually (Savanna Agricultural Research Institute, 2012; Ndamani and Watanabe, 2014). The rains start in May and end in October with the highest rainfall occurring in August and September. The rest of the year (November to May) are dry with a small number of scattered precipitations in November (Savanna Agricultural Research Institute, 2012; Ndamani and Watanabe, 2014). The first experiment included 60 medium duration groundnut genotypes (Table 1) selected from the African Groundnut Germplasm Collection (AGGC) for leaf spot resistant and yield phenotyping. The medium duration groundnut genotypes complete their life cycle within 100–120 days after sowing. A 6 × 10 rectangular alpha lattice design with three replications was used. Each replication contained six (Balota and Oakes, 2017) blocks with 10 single row plots of 2 m length in each block. The second experiment consisted of 192 short duration groundnut genotypes (Supplementary Table 1) selected from the AGGC also for leaf spot resistant and yield screening. Short duration groundnut genotypes complete their life cycle within 85–100 days after sowing. The genotypes were arranged in an 8 × 24 rectangular alpha lattice design with three replications. Each replication contained eight (Denwar et al., 2021) blocks with 24 single row plots of 2 m length in each block.

**Table 1:** List and countries of origin for 60 medium duration groundnut genotypes used for leaf spot resistant and yield phenotyping.

Number	Genotype	Country	Number	Genotype	Country
1	CHINESE	Ghana	31	MZG-ICGV-SM 03530:201909	Mozambique
2	GhaII-YENYAWOSO:201909	Ghana	32	MZG-PAN-09001:201909	Mozambique
3	ICGV 99247	Ghana	33	MZG-ICGV-SM 01513:201909	Mozambique
4	Gha-Nakpanduri 1:201909	Ghana	34	MZG-PAN-13006:201909	Mozambique
5	Gha-ICGV 07286:201909	Ghana	35	Nig-TAIMAN-9:201909	Niger
6	Gha-ICGV 15017:201909	Ghana	36	Nig-ICGVIS 07957:201909	Niger
7	Gha-ICGV-IS 13081:201909	Ghana	37	Nig-ICGVIS 79103:201909	Niger
8	GhaII-ICGV-91287:201909	Ghana	38	Nig-T-DT2-2016:201909	Niger
9	GhaII-ICGV-13009:201909	Ghana	39	Nig-ICGVSM 99502:201909	Niger
10	Gha-ICGV 00005:201909	Ghana	40	Nig-ICGV 87003:201909	Niger
11	Mwi-ICGV SM 5521:201909	Malawi	41	Nig-ICGVIS 07997:201909	Niger
12	Mwi-ICGV SM 99594:201909	Malawi	42	Nig-T-EM1-2016:201909	Niger
13	Mwi-Baka:201909	Malawi	43	Nig-ICGV 91324:201909	Niger
14	Mwi-ICGV-SM 03519:201909	Malawi	44	Nig-ICGVIS 07890:201909	Niger
15	Mwi-ICGV SM 08528:201909	Malawi	45	Sen-ICGV 96894:201909	Senegal
16	Mwi-ICG 14788:201909	Malawi	46	Sen-SERENUT 10R:201909	Senegal
17	Mwi-ICGV SM 09524:201909	Malawi	47	Sen-Fleur 11:201909	Senegal
18	Mwi-ICGV SM 07533:201909	Malawi	48	Tog-HG08:201909	Togo
19	Mwi-ICG 6057:201909	Malawi	49	Tog-HG98:201909	Togo
20	Mwi-CNG 1545:201909	Malawi	50	Tog-HG07:201909	Togo
21	Mwi-ICGV-SM 08565:201909	Malawi	51	Tog-HG65:201909	Togo
22	Mal-ICIAR 19 BT:201909	Mali	52	Oug-ICGV SM 06518:201909	Uganda
23	Mal-ICGV 86015:201909	Mali	53	Oug-ICGV SM 05650:201909	Uganda
24	Mal-ICGVIS 13825:201909	Mali	54	Oug-ICGV SM 08577:201909	Uganda
25	Mal-ICGV 86024:201909	Mali	55	Oug-ICGV SM 03590:201909	Uganda
26	Mal-ICG 81:201909	Mali	56	Oug-KadonokhoX3590 Tan:201909	Uganda
27	Mal-ICGVIS 07947:201909	Mali	57	Oug-AWI 0802 RED UG:201909	Uganda
28	Mal-86,124:201909	Mali	58	Oug-ICGV SM 99555:201909	Uganda
29	MZG-JL-24:201909	Mozambique	59	Oug-ICGV SM 07593:201909	Uganda
30	MZG-ICGV-SM 03520:201909	Mozambique	60	Zam-ICGV-SM-06637:201909	Zambia

### Data collection

#### Conventional measurements of LSD and yield

**Figure 1:**
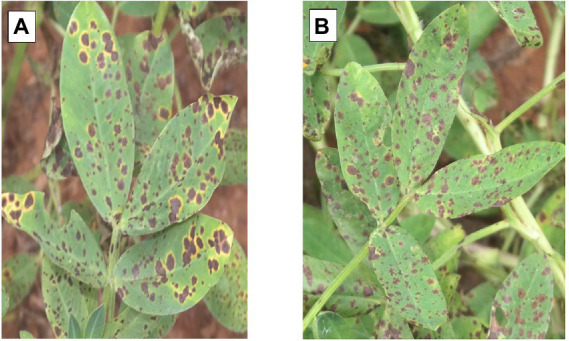
Lesions of ELS and LLS on infected leaves surface, represented by **(A,B)**, respectively.

**Figure 2:**
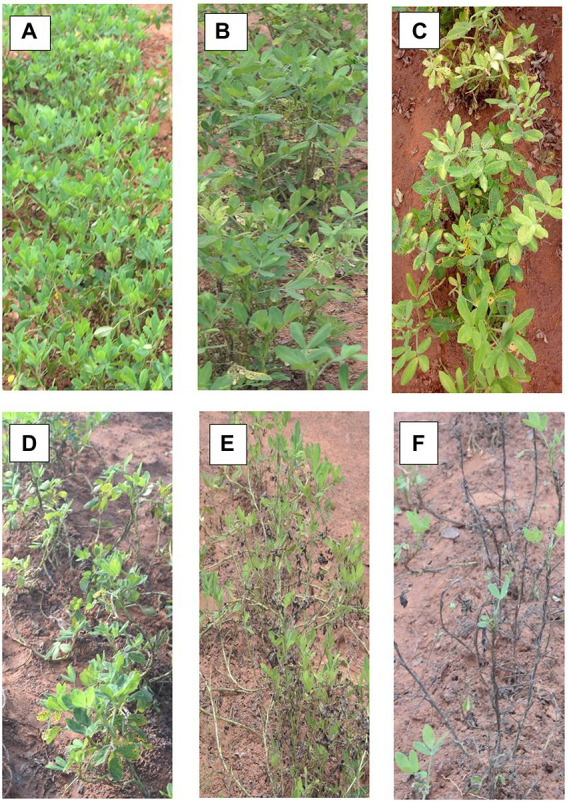
Examples of severity levels of leaf spot diseases used for visual rating of groundnut plots in this experiment [**A–F** represent scores 1, 3, 5, 6, 7, and 9, respectively, on the leaf spot scale by Subrahmanyam et al., 1995].

Both ELS and LLS mostly occur together in Ghana. An effort was made to distinguish ELS and LLS. It was possible to distinguish the two diseases easily because of the physical appearance of their spots (Figure 1). Symptoms of ELS are dark brown, yellow halo and sub-secular lesions on groundnut leaves whiles symptoms of LLS are darker, more circular lesions on the leaves and usually without yellow halo (Tshilenge-Lukanda et al., 2012). The severity of ELS and LLS infections was scored at 70, 80, 85 and 95 days after planting (DAP) based on their unique symptoms using the scale described by Subrahmanyam et al. (1995; Figure 2; Table 2). Genotypes with leaf spot scores from 1 to 3 were suggested to be resistant, genotypes scoring 4 to 6 were regarded as moderately resistant, and genotypes scoring 7 and above were considered susceptible (Gaikpa et al., 2021). Calculation of Area Under The Disease Progress Curve (AUDPC) was done for ELS and LLS from the severity scores of each plot using the formula: $AUDPC=\overset{a}{\underset{i=1}{\sum }}\left[\left\{\frac{y_{i}+y_{i+1}}{2}\right\}x\left(t_{i+1}-t_{i}\right)\right]$ , where Yi is the level of disease severity score at a point in time, *t*(*i* + 1)-ti is the number of days between two successive scores (Shaner and Finney, 1977).

**Table 2:** Leaf spot severity rating scale used in this study [from Subrahmanyam et al. (1995)].

Score	Leaf spot disease
1	No disease
2	A few, small necrotic spots on older leaves
3	Small spots, mainly on older leaves; sparse sporulation
4	Many spots, mostly on lower and middle leaves; disease evident
5	Spots easily seen on lower and middle leaves; moderate sporulation; yellowing and defoliation of some lower leaves
6	As for rating 5, but spots sporulating heavily
7	Disease easily seen from a distance; spots all over the plant; defoliation of lower and middle leaves
8	As for rating 7, but heavy defoliation
9	Plants severely affected; 50%–100% defoliation

Observations were recorded on the number of pods per plant (Pods/PLT), i.e., at the physiological maturity, pods obtained from five harvested plants were individually counted and averaged. Pod weight per plant (PW/PLT) was also performed at the physiological maturity, when pods from five plants were dug manually and hand-stripped, cleaned from soil, then air dried to constant weight, and pod weight was taken using an electronic scale (KERN^®^, PCB 10000–1; Balingen, Germany).

#### Red-green-blue imaging

The images were captured on the same days as the visual LSD ratings. A Samsung Galaxy NX300 digital camera that captures 20.3 mega-pixels was used to take images of individual plots. The camera was held horizontally in landscape mode over the plots at an angle of 90° and kept at a height of 80 cm above the plant canopy for all the imaging whiles facing the sun to avoid any shadow on the pictures. The camera was set to the “auto” mode to allow automatic adjustments for sharpness, brightness and hue (H) depending on the light available. Green area (GA = H 60–120°), greener area (GGA = H 80–120°), H angle, and crop senescence index [CSI = (100*(GA-GGA)/GA); Gracia-Romero et al., 2018] were extracted using Breedpix 2.0 option from the CIMMYT maize scanner 1.16 plugin (http://github.com/george-haddad/CIMMYT open software; Copyright 2015 Shawn Carlisle Kefauver, University of Barcelona); produced as part of Image J/Fiji (open source software; http://fiji.sc/Fiji; Schindelin et al., 2012; Rueden and Eliceiri, 2017). Both GA and GGA measures the number of green pixels on an image. However, the GGA removes green tones that are yellowish from and, accordingly, differentiates leaf senescence and active photosynthetic biomass more accurately.

### Data analysis

Data analysis was performed using the mixed linear model with R statistical program, version 4.0. 2 (R Core Team, 2018). This was carried out to identify the variability among genotypes for particular traits. Pearson correlation was computed and visualised among the parameters using the Agricolae package in R (Wei et al., 2017) to determine the correlation among the studied parameters.

#### Genotypic and phenotypic variances and coefficients of variation

##### Genotypic variance

Estimation of Genotypic Variance (VG) was done using the formula: $VG=\frac{\left(\mathrm{MSG}-\mathrm{MSE}\right)}{r}$ , where MSG is the mean squared of genotype, MSE is the mean squared of the residual (error) and r is the number of replications (Walle et al., 2014; Oteng-Frimpong et al., 2017).

##### Phenotypic variance

Calculation of phenotypic variance (VP) was done using the formula: $\;VP=\mathrm{VG}+\frac{\mathrm{MSE}}{r}$ , where VG is the genotypic variance, MSE is the mean squared of the residual (error) and r is the number of replications (Walle et al., 2014; Oteng-Frimpong et al., 2017).

##### Genotypic coefficient of variation

The formula $\mathrm{Genotypic coefficient of variation}\;\left(GCV\right)=\frac{\sqrt{\mathrm{VG}}}{X}\times 100$ was used to calculate genotypic coefficient of variation with x representing the grand mean of the trait in question (Walle et al., 2014; Oteng-Frimpong et al., 2017). GCV values were categorized as low for less than 10%, moderate for between 10 and 20% and more than 20% as high (Deshmukh et al., 1986).

##### Phenotypic coefficient of variation

Calculation of phenotypic coefficient of variation (PCV) was also carried out using the formula $PCV=\frac{\sqrt{\mathrm{VP}}}{X}\times 100$ , where x is the grand mean of the trait of interest (Walle et al., 2014; Oteng-Frimpong et al., 2017). PCV values were categorized as low for less than 10%, moderate for between 10% and 20% and more than 20% as high (Deshmukh et al., 1986).

#### Estimated broad sense heritability (H2)

Estimation of broad sense heritability was done using the formula $H2=\frac{\mathrm{VG}}{\mathrm{VP}}$ , where VG and VP, respectively, represents the genotypic and phenotypic variances (Allard, 1999). Broad sense heritability was categorized as low for less than 30%, medium for 31%–60% and 61% and above as high (Johnson et al., 1955).

#### Expected genetic advance

Calculation of expected genetic advance (EGA) was performed using the formula: GA = (K) бP H2 where GA is the expected genetic advance, K is the selection differential (2.06 at 5% selection intensity) and бA is the phenotypic standard deviation (Shukla et al., 2006).

#### Genetic advance as percentage

The genetic advance as percentage (GAM) the of mean was calculated as: $\mathrm{GAM}=\left(\frac{\mathrm{GA}}{X}\right)\times 100$ (Shukla et al., 2006). GAM was categorized as low for less than 10%, moderate for between 10 and 20% and more than 20% as high (Johnson et al., 1955).

## Results

### ELS and LLS reaction

The area under disease progress curve was used to understand the incidence and progression of ELS and LLS diseases among the groundnut genotypes using leaf spot severity scores taken at 70, 80, 85, and 95 DAP. However, the best associations with the traditional measurements were at 95 DAP, for which we primarily present here the data recorded at 95 DAP. The genotypes exhibited different levels of resistance to ELS and LLS diseases. For the medium duration population, the area under disease progress curve for ELS_AUDPC ranged from 32 for genotype 73–33 to 73 for Sen-DOK IT with a mean of 57 (Table 3). In the case of LLS_AUDPC, 73–33 again obtained the least score of 54 while genotype Oug-ICGV SM 06525 obtained the highest score of 145 with 106 as the mean of all genotypes. The results for the early duration population indicated that the genotype Mal-ICGV 02271:201909 had the lowest AUDPC score of 39 while Nig-ICGV 91324:201909 scored 66, which was the highest value (Supplementary Table 2). The mean AUDPC score for ELS among the genotypes was 58. Genotype Sen-SERENUT 10R:201909 scored 111 showing the lowest AUDPC for LLS, and Nig-TX903838:20190 had the highest AUDPC score of 204. The mean AUDPC score for LLS for all genotypes was 167.

**Table 3:** Genotypic response of different RGB-image method and conventionally rated early and late leaf spot (ELS, LLS) in groundnut at 95 days after planting.

Genotype	CSI_95	GA_95	GGA_95	Hue_95	PW/PLT (g)	Pods/plant	ELS_AUDPC	LLS_AUDPC
73–33	20.6	0.45	0.35	53.45	10.8	15.4	32.3	54
Gha-GAF 1665:201909	20.6	0.72	0.60	80.38	10.2	16.4	45.1	82
Gha-GAF 1723:201909	27.6	0.54	0.40	67.37	13.5	18.5	51.2	82
Gha-ICGV 07390:201909	17.6	0.54	0.46	63.68	14.8	18.6	53.1	96
Gha-ICGV 15008:201909	20.2	0.22	0.18	33.23	6.4	11.2	53.7	129
Gha-ICGV 15033:201909	22.8	0.35	0.27	45.95	9.1	15.0	52.9	104
GhaII-AP-NK-9-13:201909	26.5	0.31	0.24	44.81	8.6	13.7	68.6	113
GhaII-AZIVIVI:201909	19.9	0.51	0.42	59.90	10.5	14.2	52.5	71
GhaII-ICGV-13045:201909	18.0	0.35	0.30	52.80	10.0	11.9	54.8	132
GhaII-ICGV-13998:201909	30.9	0.33	0.23	46.95	9.5	13.2	67.0	109
GhaII-ICGV 03331:201909	44.2	0.26	0.13	39.14	5.6	11.0	64.1	117
GhaII-IVG 7867:201909	32.5	0.41	0.28	57.50	8.6	13.8	63.0	103
GhaII-NUMEX 05:201909	26.0	0.38	0.27	51.15	9.1	12.4	62.4	113
GhaII-SHITAOCHI:201909	26.2	0.22	0.17	34.78	9.0	12.6	64.1	127
Mal-ICG 14630:201909	29.7	0.38	0.26	49.54	5.5	10.5	62.8	110
Mal-ICGV 01258:201909	40.2	0.28	0.18	41.76	13.6	15.4	61.8	124
Mal-ICGV 08656:201909	24.0	0.47	0.36	59.01	10.0	12.7	56.6	111
Mal-ICGVIS 13835:201909	17.1	0.40	0.34	57.20	11.4	14.0	56.8	115
Mal-ICGVIS 141120:201909	23.3	0.36	0.29	48.23	12.3	13.2	69.4	121
Mwi-CG 7:201909	22.3	0.55	0.43	68.12	11.0	12.7	54.1	95
Mwi-ICG 14705:201909	20.1	0.35	0.28	49.25	10.4	15.0	56.6	109
Mwi-ICGV-SM 01711:201909	22.7	0.52	0.41	63.44	12.8	15.0	44.1	98
Mwi-ICGV-SM 01721:201909	17.2	0.35	0.29	44.01	10.5	12.7	44.8	93
Mwi-ICGV SM 07512:201909	24.1	0.41	0.31	55.24	11.1	13.1	55.0	136
Mwi-ICGV SM 1276:201909	21.3	0.34	0.28	47.20	12.2	14.7	63.0	122
MZG-ICGV-SM 01731:201909	25.2	0.49	0.37	64.05	8.5	12.8	64.2	108
MZG-Local 1:201909	20.7	0.58	0.47	68.94	9.3	14.7	52.9	96
MZG-MTP 14001:201909	25.6	0.35	0.27	49.52	10.2	10.9	63.8	115
MZG-NMP 14003:201909	19.6	0.55	0.45	65.20	9.8	12.0	42.2	92
Oug-BOUNDUCK UG:201909	30.1	0.55	0.38	67.19	7.5	11.1	59.0	108
Oug-ICGV 15021:201909	24.8	0.48	0.36	60.16	15.1	16.9	53.1	93
Oug-ICGV 15025:201909	24.6	0.24	0.18	33.96	7.7	13.0	57.2	110
Oug-ICGV 90099:201909	25.8	0.62	0.44	68.58	14.6	16.9	46.0	77
Oug-ICGV SM 02724	18.6	0.58	0.48	67.72	12.5	13.8	50.2	95
Oug-ICGV SM 06525:201909	27.9	0.25	0.18	37.31	10.2	15.7	57.3	145
Oug-ICGV SM 07510:201909	31.0	0.32	0.22	46.83	13.3	13.1	57.9	122
Oug-ICGV SM 10034:201909	20.2	0.63	0.52	75.58	14.9	16.0	53.7	93
Oug-ICGV SM 15583:201909	25.4	0.58	0.44	70.14	12.1	14.5	58.3	101
Oug-KAYOBA X 02501 UG	38.8	0.25	0.17	40.36	10.4	12.9	62.6	134
Oug-SERENUT 11 T UG:201909	38.7	0.61	0.35	65.12	9.7	13.4	58.8	95
Oug-SERENUT 9 T UG:201909	26.1	0.65	0.48	73.16	11.5	14.9	54.0	87
Oug-SGV 0023 UG:201909	20.2	0.66	0.54	78.53	11.7	14.4	55.6	90
Oug-SGV 0062 UG:201909	22.2	0.64	0.51	70.48	14.0	18.4	49.9	91
Oug-SGV 07002 UG:201909	20.6	0.62	0.51	76.39	14.5	14.5	51.1	82
Sen-69-101:201909	20.7	0.64	0.51	71.80	8.9	13.3	50.2	88
Sen-DOGO_Chin1:201909	34.9	0.23	0.14	36.68	7.8	10.5	68.6	106
Sen-DOGO_Chin4:201909	28.4	0.39	0.29	51.88	8.0	11.0	65.8	118
Sen-DOK IT:201909	24.2	0.23	0.18	30.19	7.3	11.6	72.8	130
Sen-HUAYU 33:201909	22.8	0.20	0.17	28.70	6.9	9.6	70.0	131
Sen-Souleye Badiane:201909	24.0	0.12	0.10	21.79	8.1	11.9	71.1	122
Tog-HG09:201909	25.6	0.28	0.22	42.80	7.8	11.6	70.3	129
Tog-HG100:201909	21.4	0.41	0.33	51.70	10.1	14.5	50.7	119
Tog-HG91:201909	24.6	0.27	0.21	38.34	9.3	12.7	59.7	120
Zam-CHARLIMBANA:201909	28.7	0.53	0.37	63.24	11.4	12.0	52.7	94
Zam-ICG-13099:201909	21.1	0.42	0.33	54.13	10.5	14.1	53.0	108
Zam-ICGV-SM-01514:201909	29.7	0.21	0.16	33.42	10.1	12.6	72.0	109
Zam-ICGV-SM-07599:201909	19.9	0.67	0.54	73.00	17.5	18.2	45.1	85
Zam-ICGV-SM-93522:201909	27.8	0.33	0.24	45.05	8.8	12.2	60.4	107
Zam-MGV-6:201909	23.5	0.54	0.42	64.05	10.9	13.1	46.8	94
Zam-MGV-8:201909	18.6	0.57	0.47	70.62	12.2	13.6	43.6	85
**MEAN**	**25.0**	**0.43**	**0.33**	**54.51**	**10.5**	**13.7**	**56.8**	**106**
**MIN**	**17.1**	**0.12**	**0.10**	**21.79**	**5.5**	**9.6**	**32.3**	**54**
**MAX**	**44.2**	**0.72**	**0.60**	**80.38**	**17.5**	**18.6**	**72.8**	**145**

Pod weight per plant and the number of pods per plant were taken at the physiological maturity. CSI, crop senescence index; GA, green area; GGA, greener area; Hue, ratio of green and greener area; PW/PLT, pod weight per plant; Pods/plant, number of pods per plant; ELS_AUDPC and LLS_AUDPC, area under disease progress curve for early and late leaf spot, respectively. The bold values represent the means, minimum and maximum values recorded for each trait.

### Yield

Genotypic differences were observed for the pod weight plant-1 (PW/PLT) and number of pods plant-1 (Pods/PLT). Among the genotypes, Mal-ICG 14630 had the lowest PW/PLT of 5.5 g, and Zam-ICGV-SM-07599 the highest of 17.5 g. The population mean was 10.5 g plant-1 (Table 3). For the Pods/PLT, in the medium duration population, genotypes Sen-HUAYU 33 and Sen-DOGO_Chin 1 had 10 pods plant-1as the lowest value, while Gha-GAF 1723 and Gha-ICGV 07390 had 19, the highest number of pods; 14 pods plant-1 was the mean of all genotypes. Among the early duration population, Oug-DOK 1 RED UG:201909 had the lowest PW/PLT of 5.1 g, whiles Gha-ICGV-IS 13144:201909 exhibited the highest of 18.9 g; the population mean was 8.7 g plant-1 (Supplementary Table 2). Oug-DOK 1 RED UG:201909 also produced the least number of pods, 8 pods plant-1, and Mal-ICGVIS 13827:201909 obtained the highest number of 21 pods plant-1. The population mean was 12 pods plant-1.

### Variance components, coefficient of variation, and broad sense heritability

Genotypic (Ϭ^2^g) and phenotypic (Ϭ^2^p) variance and coefficient of variation (GCV, FCV), broad sense heritability (H2), expected genetic advance (EGA) and genetic advance as percentage of the mean (GAM) for the traits estimated in this work are presented in Tables 3, 4 for both, the medium duration and early duration populations. For the medium duration population, the values for Ϭ^2^g were in the range of 0.01 for GAA at 70 DAP (GGA_70) to 353.1 for LLS_AUDPC whiles values for Ϭ^2^p were in the range of 0.01 for GGA_70 to 384.1 for LLS_AUDPC (Table 4). The values for GCV ranged from 11.7% for hue angle at 70 DAP (Hue-70) to 40.3% for GAA at 95 DAP (GGA_95), while PCV values ranged from 13% for Hue_70 to 42.7% for GGA_95. Estimated broad sense heritability ranged from 53.5% for Pods/PLT to 93% for GA at 85 DAP (GA_85). EGA values were in the range of 0.20 for GGA_70 to 37.1 for LLS_AUDPC. GAM values ranged from 21.5 for Hue_70 to 78.4 for GGA_95. Results for the early duration population were similar with the medium duration population. For example, Ϭ^2^g ranged from 0.004 for GGA_70 to 299.5 for LLS_AUDPC and Ϭ^2^p from 0.006 for GGA_70 to 353.2 for LLS_AUDPC (Table 5). GCV values started from 9.2% for ELS_AUDPC to 38% for GGA_95 while PCV values were in the range of 11.1 to 45.3% for PW/PLT. Broad sense heritability values ranged from 29.1% for the CSI at 95 DAP (CSI_95) to 84.8% for LLS_AUDPC. Values for EGA were in the range of 0.1 for GGA_70 to 32.8 for LLS_AUDPC. GAM values ranged from 15.4 for CSI at 95 DAP (CSI_95) to 66.3 for GGA_95.

**Table 4:** Genotypic and phenotypic variance, genotypic and phenotypic coefficient of variation, broad sense heritability, expected genetic advance and expected genetic advance as percentage of the mean for RGB-image method and conventionally measured traits on the medium duration population.

Trait	Ϭ^2^g	Ϭ^2^p	GCV (%)	PCV (%)	H^2^ (%)	EGA	GAM
GA_70	0.01	0.01	16.34	17.59	86.23	0.21	31.25
GGA_70	0.01	0.01	18.11	19.35	87.65	0.20	34.93
Hue_70	73.34	91.50	11.65	13.01	80.15	15.79	21.48
CSI_70	14.03	19.00	23.56	27.42	73.82	6.63	41.69
GA_80	0.02	0.02	23.16	24.67	88.13	0.26	44.79
GGA_80	0.02	0.02	25.84	27.35	89.25	0.25	50.29
Hue_80	116.99	140.63	16.08	17.63	83.19	20.32	30.22
CSI_80	18.53	22.59	24.40	26.95	82.01	8.03	45.52
GA_85	0.02	0.03	30.41	31.53	93.03	0.31	60.42
GGA_85	0.02	0.02	33.18	34.50	92.52	0.26	65.75
Hue_85	164.61	180.58	21.40	22.42	91.15	25.23	42.10
CSI_85	25.32	36.36	20.54	24.62	69.62	8.65	35.31
GA_95	0.03	0.03	37.12	39.07	90.28	0.31	72.65
GGA_95	0.02	0.02	40.33	42.72	89.13	0.26	78.44
Hue_95	231.67	258.42	27.92	29.49	89.65	29.69	54.46
CSI_95	48.23	66.52	27.83	32.69	72.50	12.18	48.82
ELS_AUDPC	86.89	102.71	16.40	17.83	84.60	17.66	31.07
LLS_AUDPC	353.06	384.14	17.77	18.54	91.91	37.11	35.09
Pods/PLT	8.32	15.57	21.12	28.88	53.47	4.35	31.81
PW/PLT(g)	9.10	12.83	28.76	34.14	70.95	5.23	49.89

Ϭ^2^g, genotypic variance; Ϭ^2^p, phenotypic variance; H^2^, broad sense heritability; GCV, genotypic coefficient of variation; PCV, phenotypic coefficient of variation; EGA, expected genetic advance and GAM, genetic advance as percentage of the mean; GA_70, GA_80, GA_85, and GA_95, green area at 70, 80, 85 and 95 days after planting (DAP); GGA_70, GGA_80, GGA_85, and GGA_95, greener area at 70, 80, 85, and 95 DAP; Hue_70, Hue_80, Hue_85, and Hue_95, hue angle at 70, 80, 85, and 95 DAP; CSI_70, CSI_80, CSI_85, and CSI_95, crop senescence index at 70, 80, 85, and 95 DAP, respectively. PW/PLT stands for pod weight per plant, ELS_AUDPC and LLS_AUDPC stands for area under disease progress curve for early and late leaf spot, respectively, and Pods/plant stand for number of pods per plant.

**Table 5:** Genotypic and phenotypic variance, genotypic and phenotypic coefficient of variation, broad sense heritability, expected genetic advance and expected genetic advance as percentage of mean for RGB-image method and conventionally measured traits on the early duration population.

Trait	Ϭ2g	Ϭ2p	GCV (%)	PCV (%)	H2 (%)	EGA	GAM
GA_70	0.005	0.008	14.438	17.977	64.502	0.122	23.887
GGA_70	0.004	0.006	15.023	19.192	61.275	0.099	24.225
Hue_70	50.225	84.866	11.614	15.097	59.182	11.231	18.406
CSI_70	10.427	18.783	16.383	21.989	55.512	4.956	25.145
GA_80	0.008	0.011	24.493	29.212	70.300	0.152	42.305
GGA_80	0.006	0.008	26.902	32.104	70.220	0.130	46.439
Hue_80	88.520	129.127	19.150	23.129	68.552	16.047	32.663
CSI_80	24.054	38.499	20.478	25.907	62.479	7.986	33.344
GA_85	0.009	0.011	30.038	34.531	75.672	0.167	53.828
GGA_85	0.006	0.008	33.767	38.777	75.831	0.139	60.574
Hue_85	68.904	96.952	20.070	23.807	71.070	14.416	34.854
CSI_85	32.510	51.710	21.696	27.363	62.870	9.313	35.438
GA_95	0.007	0.010	36.493	43.236	71.241	0.146	63.452
GGA_95	0.005	0.007	37.957	44.749	71.947	0.126	66.324
Hue_95	118.348	185.746	35.138	44.021	63.715	17.888	57.779
CSI_95	7.236	24.862	13.895	25.755	29.105	2.990	15.442
ELS_AUDPC	27.97	41.060	9.200	11.140	68.110	8.990	15.630
LLS_AUDPC	299.500	353.200	10.350	11.240	84.800	32.830	19.640
PW/PLT(g)	7.030	15.690	30.330	45.320	44.780	3.650	41.810
Pods/plant	6.620	20.160	22.370	39.040	32.830	3.040	26.400

Ϭ^2^g, genotypic variance; Ϭ^2^p, phenotypic variance; H^2^, broad sense heritability; GCV, genotypic coefficient of variation; PCV, phenotypic coefficient of variation; EGA, expected genetic advance; GAM, genetic advance as percentage of the mean; GA_70, GA_80, GA_85, and GA_95, green area at 70, 80, 85, and 95 DAP; GGA_70, GGA_80, GGA_85, and GGA_95, greener area at 70, 80, 85, and 95 DAP; Hue_70, Hue_80, Hue_85, and Hue_95, hue angle at 70, 80, 85, and 95 DAP respectively; CSI_70, CSI_80, CSI_85, and CSI_95, crop senescence index at 70, 80, 85, and 95 DAP. PW/PLT stands for pod weight per plant, ELS_AUDPC and LLS_AUDPC stands for area under disease progress curve for early and late leaf spot, respectively, and Pods/plant stand for number of pods per plant.

### Association between studied traits

The Pearson correlation matrix was employed to assess the relationship between the RGB-image method and conventionally measured traits for both, the training and validation populations. There were significant correlations (*p* < 0.05) among the parameters studied. The analysis showed a negative linear association between the RGB-image method traits (GA_85, GGA_85 and Hue_85) and the LSD scores (ELS_AUDPC and LLS_AUDPC) for both populations (Tables 6, 7), i.e., a smaller number of green pixels on the image corresponded to more diseased plots. Not surprising, CSI_85 exhibited a significant positive association with the ELS_AUDPC and LLS_AUDPC also for both populations, i.e., more senescence for more diseased plots. For the medium duration population, significant correlations were observed for GA_85 and ELS_AUDPC (r = −0.72, *p* < 0.001), GA_85 and LLS_AUDPC (r = −0.7, p < 0.001; Table 6). GA_85 and Pods/plant (r = 0.52, *p* < 0.001), and GA_85 and PW/PLT (r = 0.62, *p* < 0.001), GGA_85 also showed significant associations with ELS_AUDPC (r = −0.74, *p* < 0.001), LLS_AUDPC (r = −0.68, *p* < 0.001), Pods/plant (r = 0.56, *p* < 0.001), and PW/PLT (r = 0.66, *p* < 0.001). For the early duration population, significant correlations were observed between GA_85 with LLS_AUDPC (r = −0.66, *p* < 0.001) ELS_AUDPC (r = −0.45, *p* < 0.001), and PW/PLT (r = 0.23, *p* < 0.01; Table 7). GGA_95 also exhibited significant associations with ELS_AUDPC (r = −0.49, *p* < 0.001), LLS_AUDPC (r = −0.69, *p* < 0.001), and PW/PLT (r = 0.25, *p* < 0.01). RGB-image methodRGB-image method.

**Table 6:** Correlations among RGB-image and conventionally measured traits for the medium duration population at 85 D.A.P.

	ELS_85	LLS_85	ELS_AUDPC	LLS_AUDPC	PW/PLT (g)	Pods/plant
GA_85	−0.74^***^	−0.5^***^	−0.72^***^	−0.7^***^	0.62^***^	0.52^***^
GGA_85	−0.75^***^	−0.48^***^	−0.74^***^	−0.68^***^	0.66^***^	0.56^***^
Hue_85	−0.7^***^	−0.43^**^	−0.67^***^	−0.63^***^	0.63^***^	0.51^***^
CSI_85	0.42^**^	0.19	0.47^***^	0.25^*^	−0.49^***^	−0.37^**^

*N* = 60, PW/PLT stands for pod weight per plant; ELS_AUDPC and LLS_AUDPC stands for area under disease progress curve for early and late leaf spot, respectively; CSI stands for crop stress index; GA and GGA stand for green area and greener area of vegetation, respectively; Pods/plant represents number of pods per plant; and Hue angle is the angle (°) of the color in a 360°circle from red back to red. *p < 0.05, **p < 0.01 and ***p < 0.001.

**Table 7:** Correlations among RGB-image and manually measured traits for early duration population at 85 D.A.P.

	ELS_85	Pods/PLT	PW/PLT	ELS_AUDPC	LLS_AUDPC	LLS_85
CSI_85	0.4^***^	−0.16^*^	−0.17^*^	0.34^***^	0.35^***^	0.36^***^
GA_85	−0.74^***^	0.17^*^	0.23^**^	−0.45^***^	−0.66^***^	−0.56^***^
GGA_85	−0.75^***^	0.19^**^	0.25^**^	−0.49^***^	−0.69^***^	−0.58^***^
Hue_85	−0.69^***^	0.16^*^	0.21^**^	−0.4^***^	−0.62^***^	−0.5^***^

*N* = 192, PW/PLT stands for pod weight per plant; ELS_AUDPC and LLS_AUDPC stands for area under disease progress curve for early and late leaf spot, respectively; CSI stands for crop stress index; GA and GGA stand for green area and greener area of vegetation, respectively; Pods/plant represents number of pods per plant; and Hue angle is the angle (°) of the color in a 360°circle from red back to red. *p < 0.05, **p < 0.01 and ***p < 0.001.

### Principal component analysis

The Principal component analysis (PCA) was used to identify the most important traits in this study. For the training population, Principal components one (PC1) and two (PC2) were those considered with the greatest contribution to the observed variability among the genotypes based on their eigenvalues (Table 8). These two principal components cumulatively contributed to 76.8% of the total variation. PC1 accounted for 65.2% of the variation with the traits GA_95 (−0.408), GGA_95 (−0.418), Hue_95 (−0.4), ELS_AUDPC (0.351) and LLS_AUDPC (0.354) having the highest contributions to the variation. PC2 contributed to 11.7% of the variation and had traits Pods/PLT (0.627) and PW/*p* (0.621) as the most important traits influencing this principal component. For the validation population, the first three principal components (PC1, PC2, and PC3) were those regarded as having a significant contribution to the total observed variation existing among the genotypes judging by their eigenvalues, and they accounted for 87.5% of the total variation (Table 8). PC1 contained GA_95 (−0.469), GGA_95 (−0.475), Hue_95 (−0.452), and LLS_AUDPC (0.404) as traits accounting for most of the variation. PC2 included Pods/PLT (−0.683) and PW/PLT (−0.657), and PC3 contained CSI_95 (0.864) as the only trait accounting for most of the variation.

**Table 8:** Loadings of the traits measured at 95 days from planting (RGB-image method and disease traits), and at the physiological maturity (pod yield per plant and the number of pods per plant) onto 8 principal components for medium duration and early duration populations.

	PC1	PC2	PC3	PC4	PC5	PC6	PC7	PC8
**Medium duration population**								
CSI_95	**0.222**	0.255	**0.864**	0.228	0.232	−0.001	−0.096	0.152
GA_95	**−0.408**	−0.188	0.273	−0.226	0.071	0.091	**−0.438**	**−0.684**
GGA_95	**−0.418**	−0.198	0.067	−0.286	0.006	0.081	**−0.431**	**0.713**
Hue_95	**−0.400**	−0.179	0.284	**−0.316**	0.138	0.042	**0.778**	0.026
ELS_AUDPC	**0.351**	0.115	0.219	**−0.608**	**−0.668**	−0.001	−0.001	−0.022
LLS_AUDPC	**0.354**	0.19	−0.183	**−0.553**	**0.662**	0.238	−0.062	−0.014
Pods/PLT	**−0.316**	**0.627**	−0.076	0.106	−0.181	**0.674**	0.048	−0.009
PW/P	**−0.317**	**0.621**	−0.077	−0.169	0.071	**−0.687**	−0.035	−0.018
Eigenvalues	**5.213**	**0.933**	**0.857**	0.508	0.264	0.202	0.016	0.006
Proportion	0.652	0.117	0.107	0.063	0.033	0.025	0.002	0.001
Cumulative Proportion	0.652	0.768	0.875	0.939	0.972	0.997	0.999	1
**Early duration population**								
CSI_95	0.172	0.095	**0.875**	−0.397	−0.148	0.027	−0.104	0.074
GA_95	**−0.469**	0.172	0.155	0.083	0.216	0.014	**−0.428**	**−0.7**
GGA_95	**−0.475**	0.157	0.059	0.116	0.232	−0.021	**−0.418**	**0.71**
Hue_95	**−0.452**	0.184	0.231	0.047	0.278	−0.008	**0.793**	0.006
ELS_AUDPC	**0.295**	−0.051	0.359	**0.88**	0.084	0.007	−0.007	0.004
LLS_AUDPC	**0.404**	−0.033	−0.029	−0.211	**0.886**	0.05	−0.052	−0.004
Pods/PLT	−0.166	**−0.683**	0.126	−0.038	0.084	**−0.693**	−0.007	−0.017
PW/P	−0.208	**−0.657**	0.089	−0.013	0.03	**0.718**	0.006	0.016
Eigenvalues	**4.053**	**1.68**	**0.982**	0.663	0.39	0.176	0.05	0.006
Proportion	0.507	0.21	0.123	0.083	0.049	0.022	0.006	0.001
Cumulative proportion	0.507	0.717	0.839	0.922	0.971	0.993	0.999	1

PC1 to PC8, principal components 1 to 8; CSI_95, crop senescence index; GA_95 at 95 days after planting, green area at 95 days after planting; GGA_95, greener area at 95 days after planting; Hue_95, ratio of green and greener area at 95 days after planting; ELS_AUPDPC and LLS_AUDPC, area under disease progress curve for ELS and LLS respectively, Pods/PLT, number of pods per plant and PW/P, pod weight per plant. The bold values represent the values for traits with the highest contributions to the variability in the various principal components (ie PC 1 to PC 8). Moreover, principal components (PCs) with bold eigenvalues are those with significant contributions to the total variability existing among the genotypes.

## Discussion

The challenge to feed the growing human population in the face of numerous constrains for the agricultural production including biotic and abiotic stresses is an uphill task. Efficient phenotyping can help breeding programs develop more rapidly productive and resistant varieties of crops. In this study, 60 medium duration and 192 short duration accessions from the AGGC were used to develop improved phenotyping approaches for the leaf spot resistance and yield in groundnut. Originated under diverse agroecological conditions, these accessions represent an important genetic resource for biotic and abiotic stress resistance, which is the key to genetic gain and crop improvement (Zanklan, 2003). Indeed, in this study, a great diversity of ELS and LLS symptoms was observed among the genotypes that could be attributed to their genetic ability to respond differently to infection by the casual pathogens (White et al., 2012). For example, genotypes 73–33, Zam-MGV-8:201909, Mwi-ICGV-SM 01711:201909, Zam-ICGV-SM-07599:201909, Gha-GAF 1723:201909, Oug-ICGV 90099:201909, Mal-ICGV 02271:201909, Sen-SERENUT 10R:201909, and GhaII-AZIVIVI:201909 were moderately tolerant to the leaf spot diseases. Genotypes Gha-ICGV 07390:201909, Gha-GAF 1723:201909, Zam-ICGV-SM-07599:201909 and Oug-ICGV 15021:201909 exhibited high values for PW/PLT and Pods/PLT, and three out of four genotypes with good yield traits were also resistant to the LSD. Further assessment of these genotypes for use in crosses or release will go a long way to boost groundnut production in Northern Ghana. Fungicide application, which is a commonly used method in developed countries, is not applicable in Ghana because the farmers cannot afford their high cost (Denwar et al., 2021). Growing resistant varieties is the only option in controlling these diseases in developing countries. Furthermore, water pollution is minimized when farmers use less chemicals, in particular in fields neighboring water bodies where chemicals can be washed in by heavy rains following immediately after their application.

The estimated phenotypic coefficients of variation were higher compared to the genotypic coefficients of variation for the studied parameters. This observation was not different from the findings of Oteng-Frimpong et al. (2017). However, the differences between GCV% and the corresponding PCV% were narrow, suggesting lesser influence of the environmental factors in the expression of these parameters implying that variability was largely due to genetic effects (Tsegaye et al., 2007; Okwuagwu et al., 2008; Singh et al., 2013). The estimation of heritability gives information about the portion of variation which can be transferred from parent to the subsequent generation (Visscher et al., 2008). Effective exploitation of genotypic variability through selection is based on individual traits’ heritability (Bilgin et al., 2010). The high broad sense heritability of the RGB-image method indices in this study is an indication that these indices will be best to select for, as the environment has a minimal influence on their expression (Oteng-Frimpong et al., 2017). It is also worth mentioning that medium heritability for Pods/PLT and high heritability for PW/PLT were observed for the training population, suggesting high possibility of improving these traits. This observation was not surprising to us given the diverse genetic background of the genotypes. Information about heritability alone is not enough to make conclusion whether selection will lead to improvement since it does not give enough information as to the rate of genetic gain that can be obtained through selection (Singh et al., 2013). The high heritability and high genetic advance observed for some of the studied traits indicates additive gene action suggesting phenotypic selection for such traits is highly possible. For example, Hue_95 and CSI_95 with both high heritability and expected genetic advance are best selection targets for improvement of leaf spot resistance in breeding programs. High genetic advance as percentage of the mean from selecting the best 5% of the genotypes coupled with high broad sense heritability recorded for most of the RGB-image method traits in this study indicates additive gene action (Govindaraj et al., 2011; Kant et al., 2012).

Correlation analysis gives important information about the association between traits (Owusu et al., 2018; Ajayi and Gbadamosi, 2020; Mofokeng et al., 2020). Pearson correlation matrix was employed to assess the relationship between the RGB-image method and conventionally measured traits for both training and validation populations. There were significant correlations (*p* < 0.05) among the studied parameters indicating that RGB-image method has the potential to replace or complement the conventional methods of data collection due to the easy application and less expensive nature of the technology. Findings from this research suggest potential for automatization of disease severity and yield components assessment that will enable faster data collection at multiple/relevant time points throughout the growing season. The positive correlation between GA, GGA and Hue and yield components (PW/PLT and Pods/PLT) indicates that improving GA, GGA and Hue will directly lead to improvement in yield components. The strong association between GA and LLS_AUDPC, GGA and LLS_AUDPC and Hue and LLS_AUDPC provides an opportunity for the development of LLS resistant cultivars through indirect selection. Because of polygenic nature of ELS and LLS (Younis et al., 2020), the direct/traditional selection is hindered by the environmental effect. When selecting based on GA, GGA, and Hue, however, our data indicate less environmental effect and higher heritability than for visual selection.

The PCA was used to determine genetic variability among the groundnut genotypes for both training and validation populations. PC1 contributed to 50.7% of the total variation for the training population and 65.2% for the validation population, and contrasted GA, GGA and Hue with LLS_AUDPC, ELS_AUDPC and CSI. This observation showed that genotypes that scored lower values for GA, GGA and Hue scored high values for LLS_AUDPC, ELS_AUDPC and CSI, and such genotypes should be discarded during selection. PC2, which accounted for 21% of the total variation for the training population and 11.7% for the validation population, was mainly influenced by yield components suggesting that genotypes with the highest contribution to PC2 could be targeted for yield improvement.

Significant correlations (*p* ≤ 0.001) were observed between predicted and the observed disease scores for all six prediction models. GA, GGA, and Hue angle, representing the number of green RGB-image method.

## Conclusion

This study showed the potential of using RGB-image method as a high-throughput tool for phenotyping leaf spot diseases and yield components estimation in groundnut. Fast progress in groundnut improvement could be achieved when RGB-image method traits are use as surrogate traits for selecting leaf spots resistance in groundnut breeding programs in Ghana. GA, GGA, Hue, and CSI can successfully replace or, at least, complement the conventional methods for leaf spot diseases phenotyping in groundnut. This study reveals that photogrammetric techniques were more effective at the later rather than early stages of vegetation and when disease symptoms are ample.

Genotypes 73–33, Gha-GAF 1723:201909, Zam-ICGV-SM-07599:201909, and Oug-ICGV 90099:201909 were identified as promising sources for leaf spot diseases resistance and high yield components for the groundnut breeding programs in Ghana.

## Data availability statement

The raw data supporting the conclusions of this article will be made available by the authors, without undue reservation.

## Author contributions

ES conducted the experiments, analyzed the data and wrote the manuscript. RO-F coordinated the project, provided the materials, supervised the experiments, and reviewed and edited the manuscript. YK designed the experiments, assisted in data collection and statistical analysis, review and edited the manuscript, DP, JA-D, and AM assisted in trials establishment, data analysis, reviewed and edited the manuscript. AD and KO as academic supervisors, supervised the experiments and reviewed and edited the manuscript. MB acquired the funds, coordinated the project, supervised the experiments, and reviewed and edited the manuscript. AC helped in statistical analysis, reviewed and edited the manuscript. DH and JR acquired funding for the project, reviewed and edited the manuscript. All authors contributed to the article and approved the submitted version.

## Funding

This project was funded by the USAID, Feed the Future Peanut Innovation Lab.

## Conflict of interest

AC was employed by company Bayer Crop Science.

The remaining authors declare that the research was conducted in the absence of any commercial or financial relationships that could be construed as a potential conflict of interest.

## Publisher’s note

All claims expressed in this article are solely those of the authors and do not necessarily represent those of their affiliated organizations, or those of the publisher, the editors and the reviewers. Any product that may be evaluated in this article, or claim that may be made by its manufacturer, is not guaranteed or endorsed by the publisher.
