# Supplementary material for: RGB-image method enables indirect selection for leaf spot resistance and yield estimation in a groundnut breeding program in Western Africa
Source: Front Plant Sci. 2022 Aug 4;13:957061. doi: 10.3389/fpls.2022.957061 (PMC9387199; doi:10.3389/fpls.2022.957061)
Supplement: Supplementary file 1 [file Data_Sheet_1.docx]

Supplementary table 1: List and countries of origin for 192 groundnut genotypes used to validate prediction models for leaf spot diseases and yield.

| Number | DESIGNATION | Country | Number | DESIGNATION | Country |
| --- | --- | --- | --- | --- | --- |
| 1 | CHINESE | Ghana | 97 | Nig-ICGV 91324:201909 | Niger |
| 2 | Gha-CHINESE:201909 | Ghana | 98 | Nig-ICGV 94434:201909 | Niger |
| 3 | Gha-ICGV 00005:201909 | Ghana | 99 | Nig-ICGVIS 07815:201909 | Niger |
| 4 | Gha-ICGV 07286:201909 | Ghana | 100 | Nig-ICGVIS 07890:201909 | Niger |
| 5 | Gha-ICGV 15017:201909 | Ghana | 101 | Nig-ICGVIS 07957:201909 | Niger |
| 6 | Gha-ICGV 15046:201909 | Ghana | 102 | Nig-ICGVIS 07964:201909 | Niger |
| 7 | Gha-ICGV 93305:201909 | Ghana | 103 | Nig-ICGVIS 07997:201909 | Niger |
| 8 | Gha-ICGV-IS 08837:201909 | Ghana | 104 | Nig-ICGVIS 07999:201909 | Niger |
| 9 | Gha-ICGV-IS 13081:201909 | Ghana | 105 | Nig-ICGVIS 79103:201909 | Niger |
| 10 | Gha-ICGV-IS 13110:201909 | Ghana | 106 | Nig-ICGVSM 99502:201909 | Niger |
| 11 | Gha-ICGV-IS 13144:201909 | Ghana | 107 | Nig-ICGV-SM-99506:201909 | Niger |
| 12 | GhaII-CROPS NKATIE:201909 | Ghana | 108 | Nig-ICIAR 19-BT:201909 | Niger |
| 13 | GhaII-DEHYEE:201909 | Ghana | 109 | Nig-NELSON-SPANISH:201909 | Niger |
| 14 | GhaII-ICGV-13009:201909 | Ghana | 110 | Nig-SRV1-3:201909 | Niger |
| 15 | GhaII-ICGV-13077:201909 | Ghana | 111 | Nig-T13-89:201909 | Niger |
| 16 | GhaII-ICGV-91287:201909 | Ghana | 112 | Nig-T14-89:201909 | Niger |
| 17 | GhaII-ICGV-IS-1300:201909 | Ghana | 113 | Nig-T16-89:201909 | Niger |
| 18 | GhaII-ICGV-IS-13114:201909 | Ghana | 114 | Nig-T169-83:201909 | Niger |
| 19 | GhaII-ICGV-IS-1399:201909 | Ghana | 115 | Nig-T177-83:201909 | Niger |
| 20 | GhaII-ICGV-IS-97188:201909 | Ghana | 116 | Nig-T183-83:201909 | Niger |
| 21 | GhaII-NUMEX 03:201909 | Ghana | 117 | Nig-T1-95:201909 | Niger |
| 22 | GhaII-PION:201909 | Ghana | 118 | Nig-T2-2007:201909 | Niger |
| 23 | GhaII-YENYAWOSO:201909 | Ghana | 119 | Nig-T4-2006:201909 | Niger |
| 24 | Gha-L 27:201909 | Ghana | 120 | Nig-T4-83:201909 | Niger |
| 25 | Gha-Nakpanduri 1:201909 | Ghana | 121 | Nig-T4-89:201909 | Niger |
| 26 | Gha-Nakpanduri 2:201909 | Ghana | 122 | Nig-T92-83:201909 | Niger |
| 27 | Gha-SARGV 005:201909 | Ghana | 123 | Nig-T95-83:201909 | Niger |
| 28 | Gha-White skin:201909 | Ghana | 124 | Nig-TAIMAN-9:201909 | Niger |
| 29 | YENYAWOSO | Ghana | 125 | Nig-T-DT1-2016:201909 | Niger |
| 30 | Mwi-Baka:201909 | Malawi | 126 | Nig-T-DT2-2016:201909 | Niger |
| 31 | Mwi-CG 13:201909 | Malawi | 127 | Nig-T-EM1-2016:201909 | Niger |
| 32 | Mwi-Chitala:201909 | Malawi | 128 | Nig-T-EM2-2016:201909 | Niger |
| 33 | Mwi-CNG 1545:201909 | Malawi | 129 | Nig-TM-H-94:201909 | Niger |
| 34 | Mwi-ICG 14788:201909 | Malawi | 130 | Nig-T-RT1-2016:201909 | Niger |
| 35 | Mwi-ICG 5195:201909 | Malawi | 131 | Nig-TX903654:201909 | Niger |
| 36 | Mwi-ICG 6057:201909 | Malawi | 132 | Nig-TX903796:201909 | Niger |
| 37 | Mwi-ICG 6888:201909 | Malawi | 133 | Nig-TX903838:201909 | Niger |
| 38 | Mwi-ICGV SM 07504:201909 | Malawi | 134 | Sen-55-33:201909 | Senegal |
| 39 | Mwi-ICGV SM 07532:201909 | Malawi | 135 | Sen-55-437:201909 | Senegal |
| 40 | Mwi-ICGV SM 07533:201909 | Malawi | 136 | Sen-73-30:201909 | Senegal |
| 41 | Mwi-ICGV SM 07539:201909 | Malawi | 137 | Sen-78-936:201909 | Senegal |
| 42 | Mwi-ICGV SM 07544:201909 | Malawi | 138 | Sen-Boulkouss:201909 | Senegal |
| 43 | Mwi-ICGV SM 08528:201909 | Malawi | 139 | Sen-DOGO_Chin2:201909 | Senegal |
| 44 | Mwi-ICGV SM 08533:201909 | Malawi | 140 | Sen-DOGO_Chin6:201909 | Senegal |
| 45 | Mwi-ICGV SM 09524:201909 | Malawi | 141 | Sen-DOGO_Chin8:201909 | Senegal |
| 46 | Mwi-ICGV SM 5521:201909 | Malawi | 142 | Sen-Fleur 11:201909 | Senegal |
| 47 | Mwi-ICGV SM 99594:201909 | Malawi | 143 | Sen-ICGV 96894:201909 | Senegal |
| 48 | Mwi-ICGV-SM 03519:201909 | Malawi | 144 | Sen-L4:201909 | Senegal |
| 49 | Mwi-ICGV-SM 05738:201909 | Malawi | 145 | Sen-LONGHU 1:201909 | Senegal |
| 50 | Mwi-ICGV-SM 08565:201909 | Malawi | 146 | Sen-Pungsan:201909 | Senegal |
| 51 | Mal-55-437:201909 | Mali | 147 | Sen-Schubert:201909 | Senegal |
| 52 | Mal-86124:201909 | Mali | 148 | Sen-SERENUT 10R:201909 | Senegal |
| 53 | Mal-FLEUR11:201909 | Mali | 149 | Sen-Taaru:201909 | Senegal |
| 54 | Mal-ICG 81:201909 | Mali | 150 | Tog-HG03:201909 | Togo |
| 55 | Mal-ICGV 00350:201909 | Mali | 151 | Tog-HG07:201909 | Togo |
| 56 | Mal-ICGV 02271:201909 | Mali | 152 | Tog-HG08:201909 | Togo |
| 57 | Mal-ICGV 86015:201909 | Mali | 153 | Tog-HG48:201909 | Togo |
| 58 | Mal-ICGV 86024:201909 | Mali | 154 | Tog-HG59:201909 | Togo |
| 59 | Mal-ICGV 99240:201909 | Mali | 155 | Tog-HG65:201909 | Togo |
| 60 | Mal-ICGVIS 07947:201909 | Mali | 156 | Tog-HG80:201909 | Togo |
| 61 | Mal-ICGVIS 09996:201909 | Mali | 157 | Tog-HG82:201909 | Togo |
| 62 | Mal-ICGVIS 13079:201909 | Mali | 158 | Tog-HG85:201909 | Togo |
| 63 | Mal-ICGVIS 131096:201909 | Mali | 159 | Tog-HG92:201909 | Togo |
| 64 | Mal-ICGVIS 13825:201909 | Mali | 160 | Tog-HG98:201909 | Togo |
| 65 | Mal-ICGVIS 13827:201909 | Mali | 161 | Oug-709 X VC74 RED UG:201909 | Uganda |
| 66 | Mal-ICGVIS 13863:201909 | Mali | 162 | Oug-Acholi white:201909 | Uganda |
| 67 | Mal-ICGVIS 13871:201909 | Mali | 163 | Oug-AWI 0802 RED UG:201909 | Uganda |
| 68 | Mal-ICGVIS 13910:201909 | Mali | 164 | Oug-DOK 1 RED UG:201909 | Uganda |
| 69 | Mal-ICGVIS 14849:201909 | Mali | 165 | Oug-ICGV SM 00537:201909 | Uganda |
| 70 | Mal-ICIAR 19 BT:201909 | Mali | 166 | Oug-ICGV SM 01504:201909 | Uganda |
| 71 | Mal-MWENJE:201909 | Mali | 167 | Oug-ICGV SM 03590:201909 | Uganda |
| 72 | MZG-35B:201909 | Mozambique | 168 | Oug-ICGV SM 05650:201909 | Uganda |
| 73 | MZG-75B:201909 | Mozambique | 169 | Oug-ICGV SM 05702:201909 | Uganda |
| 74 | MZG-CBA 13001:201909 | Mozambique | 170 | Oug-ICGV SM 06518:201909 | Uganda |
| 75 | MZG-ICG 12991:201909 | Mozambique | 171 | Oug-ICGV SM 07540:201909 | Uganda |
| 76 | MZG-ICGV-SM 01513:201909 | Mozambique | 172 | Oug-ICGV SM 07593:201909 | Uganda |
| 77 | MZG-ICGV-SM 03520:201909 | Mozambique | 173 | Oug-ICGV SM 08556:201909 | Uganda |
| 78 | MZG-ICGV-SM 03530:201909 | Mozambique | 174 | Oug-ICGV SM 08572:201909 | Uganda |
| 79 | MZG-ICGV-SM 08528:201909 | Mozambique | 175 | Oug-ICGV SM 08577:201909 | Uganda |
| 80 | MZG-JL-24:201909 | Mozambique | 176 | Oug-ICGV SM 08586:201909 | Uganda |
| 81 | MZG-NMP 13003:201909 | Mozambique | 177 | Oug-ICGV SM 10005:201909 | Uganda |
| 82 | MZG-PAN-09001:201909 | Mozambique | 178 | Oug-ICGV SM 95360:201909 | Uganda |
| 83 | MZG-PAN-09003:201909 | Mozambique | 179 | Oug-ICGV SM 99537:201909 | Uganda |
| 84 | MZG-PAN-09021:201909 | Mozambique | 180 | Oug-ICGV SM 99551:201909 | Uganda |
| 85 | MZG-PAN-09023:201909 | Mozambique | 181 | Oug-ICGV SM 99555:201909 | Uganda |
| 86 | MZG-PAN-13004:201909 | Mozambique | 182 | Oug-KadonokhoX3590 Tan:201909 | Uganda |
| 87 | MZG-PAN-13005:201909 | Mozambique | 183 | Oug-RED BEAUTY UG:201909 | Uganda |
| 88 | MZG-PAN-13006:201909 | Mozambique | 184 | Oug-SGV 99046 UG:201909 | Uganda |
| 89 | MZG-PAN-13011:201909 | Mozambique | 185 | Oug-SGV ER 10010 UG:201909 | Uganda |
| 90 | MZG-PAN-13014:201909 | Mozambique | 186 | Zam-12CS_121:201909 | Zambia |
| 91 | Nig-0-20:201909 | Niger | 187 | Zam-6:201909 | Zambia |
| 92 | Nig-55-437:201909 | Niger | 188 | Zam-8:201909 | Zambia |
| 93 | Nig-796:201909 | Niger | 189 | Zam-ICGV-07541:201909 | Zambia |
| 94 | Nig-ICGV 8602:201909 | Niger | 190 | Zam-ICGV-SM-06637:201909 | Zambia |
| 95 | Nig-ICGV 87003:201909 | Niger | 191 | Zam-ICGV-SM-07517:201909 | Zambia |
| 96 | Nig-ICGV 91317:201909 | Niger | 192 | Zam-KATETE:201909 | Zambia |

Supplementary table 2: Genotypic response of different photogrammetry and conventionally rated early and late leaf spot (ELS, LLS) in groundnut at 95 days after planting. Pod weight per plant and the number of pods per plant were taken at the physiological maturity

| Genotype | CSI_95 | GA_95 | GGA_95 | Hue_95 | Pods/plant | PW/PLT (g) | ELS_AUDPC | LLS_AUDPC |
| --- | --- | --- | --- | --- | --- | --- | --- | --- |
| CHINESE | 19.01 | 0.24 | 0.19 | 36.48 | 10.18 | 6.48 | 57.51 | 164.8 |
| Gha-CHINESE:201909 | 18.76 | 0.18 | 0.15 | 24.71 | 12.92 | 8.55 | 59.88 | 171.09 |
| Gha-ICGV-IS 08837:201909 | 20.10 | 0.25 | 0.20 | 34.04 | 14.30 | 12.11 | 59.17 | 163.59 |
| Gha-ICGV-IS 13081:201909 | 19.17 | 0.30 | 0.25 | 41.57 | 11.18 | 8.06 | 57.02 | 142.15 |
| Gha-ICGV-IS 13110:201909 | 19.62 | 0.25 | 0.20 | 34.46 | 9.32 | 7.65 | 63.09 | 152.62 |
| Gha-ICGV-IS 13144:201909 | 17.44 | 0.26 | 0.22 | 32.87 | 13.25 | 18.94 | 55.68 | 142.05 |
| Gha-ICGV 00005:201909 | 20.70 | 0.35 | 0.27 | 45.01 | 12.27 | 9.50 | 56.37 | 159.25 |
| Gha-ICGV 07286:201909 | 18.31 | 0.26 | 0.22 | 33.65 | 12.19 | 9.90 | 56.99 | 138.17 |
| Gha-ICGV 15017:201909 | 18.77 | 0.33 | 0.28 | 42.85 | 12.54 | 10.02 | 49.36 | 128.45 |
| Gha-ICGV 15046:201909 | 19.76 | 0.37 | 0.30 | 45.99 | 11.25 | 7.94 | 59.14 | 156.52 |
| Gha-ICGV 93305:201909 | 19.15 | 0.29 | 0.23 | 38.76 | 11.11 | 8.31 | 50.69 | 159.82 |
| Gha-L 27:201909 | 20.90 | 0.25 | 0.19 | 35.43 | 9.73 | 7.35 | 60.97 | 169.3 |
| Gha-Nakpanduri 1:201909 | 17.58 | 0.48 | 0.42 | 60.68 | 11.70 | 8.97 | 56.36 | 141.52 |
| Gha-Nakpanduri 2:201909 | 19.59 | 0.19 | 0.15 | 28.24 | 10.92 | 7.99 | 58.34 | 172.23 |
| Gha-SARGV 005:201909 | 18.59 | 0.26 | 0.22 | 34.27 | 11.64 | 8.26 | 60.4 | 172.09 |
| Gha-White skin:201909 | 18.48 | 0.20 | 0.16 | 25.69 | 11.79 | 8.82 | 55.18 | 159.78 |
| GhaII-CROPS NKATIE:201909 | 21.23 | 0.18 | 0.14 | 25.93 | 11.06 | 7.84 | 62.82 | 161.75 |
| GhaII-DEHYEE:201909 | 20.84 | 0.25 | 0.19 | 34.32 | 10.88 | 8.29 | 62.4 | 169.12 |
| GhaII-ICGV-13009:201909 | 21.95 | 0.23 | 0.18 | 33.80 | 12.87 | 9.75 | 60.99 | 159.74 |
| GhaII-ICGV-13077:201909 | 20.02 | 0.25 | 0.20 | 34.23 | 12.16 | 9.58 | 51.24 | 157.86 |
| GhaII-ICGV-91287:201909 | 21.07 | 0.25 | 0.20 | 35.28 | 11.91 | 8.86 | 63.78 | 152.03 |
| GhaII-ICGV-IS-1300:201909 | 18.73 | 0.25 | 0.21 | 33.87 | 10.50 | 7.35 | 56.39 | 158.74 |
| GhaII-ICGV-IS-13114:201909 | 17.90 | 0.35 | 0.30 | 42.92 | 13.41 | 9.35 | 52.9 | 132.62 |
| GhaII-ICGV-IS-1399:201909 | 18.29 | 0.27 | 0.23 | 33.93 | 10.46 | 7.67 | 58.58 | 162.09 |
| GhaII-ICGV-IS-97188:201909 | 18.53 | 0.24 | 0.20 | 33.06 | 12.44 | 9.45 | 61.03 | 149.57 |
| GhaII-NUMEX 03:201909 | 18.57 | 0.33 | 0.28 | 43.47 | 11.00 | 7.21 | 49.73 | 153.69 |
| GhaII-PION:201909 | 17.51 | 0.20 | 0.17 | 25.39 | 10.10 | 8.17 | 58.63 | 153.17 |
| GhaII-YENYAWOSO:201909 | 18.77 | 0.25 | 0.21 | 34.39 | 11.63 | 8.81 | 53.75 | 167.33 |
| Mal-55-437:201909 | 19.24 | 0.25 | 0.20 | 32.71 | 11.80 | 8.73 | 59.82 | 166.91 |
| Mal-86124:201909 | 18.91 | 0.22 | 0.19 | 34.75 | 11.77 | 9.16 | 61.48 | 148.96 |
| Mal-FLEUR11:201909 | 20.85 | 0.26 | 0.21 | 35.48 | 11.12 | 7.47 | 60.37 | 161.59 |
| Mal-ICG 81:201909 | 20.01 | 0.25 | 0.20 | 35.62 | 12.22 | 8.41 | 53.47 | 168.14 |
| Mal-ICGV 00350:201909 | 19.35 | 0.18 | 0.15 | 25.12 | 12.56 | 9.68 | 62.09 | 152.07 |
| Mal-ICGV 02271:201909 | 16.97 | 0.17 | 0.14 | 21.29 | 10.19 | 7.46 | 38.94 | 161.1 |
| Mal-ICGV 86015:201909 | 18.13 | 0.19 | 0.17 | 27.53 | 13.20 | 10.13 | 58.47 | 160.66 |
| Mal-ICGV 86024:201909 | 19.55 | 0.17 | 0.13 | 27.96 | 11.90 | 8.73 | 59.14 | 163.2 |
| Mal-ICGV 99240:201909 | 17.75 | 0.37 | 0.33 | 49.57 | 11.36 | 8.18 | 54.28 | 154.33 |
| Mal-ICGVIS 07947:201909 | 19.45 | 0.24 | 0.20 | 30.82 | 13.00 | 10.07 | 55.23 | 164.19 |
| Mal-ICGVIS 09996:201909 | 17.06 | 0.23 | 0.20 | 31.74 | 12.32 | 10.54 | 55.54 | 146.82 |
| Mal-ICGVIS 13079:201909 | 19.87 | 0.23 | 0.18 | 33.72 | 11.00 | 8.18 | 59.22 | 166.62 |
| Mal-ICGVIS 131096:201909 | 20.86 | 0.23 | 0.17 | 31.71 | 11.77 | 8.38 | 59.79 | 172.76 |
| Mal-ICGVIS 13825:201909 | 17.75 | 0.24 | 0.20 | 30.98 | 13.86 | 11.03 | 58.1 | 159.47 |
| Mal-ICGVIS 13827:201909 | 18.54 | 0.16 | 0.13 | 25.50 | 20.94 | 17.89 | 61.47 | 164.78 |
| Mal-ICGVIS 13863:201909 | 17.55 | 0.19 | 0.16 | 18.13 | 13.25 | 9.95 | 59.77 | 169.87 |
| Mal-ICGVIS 13871:201909 | 20.59 | 0.16 | 0.13 | 21.46 | 13.19 | 9.94 | 56.81 | 179.32 |
| Mal-ICGVIS 13910:201909 | 18.77 | 0.29 | 0.24 | 39.18 | 11.90 | 8.57 | 59.88 | 156.63 |
| Mal-ICGVIS 14849:201909 | 18.17 | 0.22 | 0.18 | 27.45 | 13.26 | 10.39 | 60.78 | 160.59 |
| Mal-ICIAR 19 BT:201909 | 18.26 | 0.28 | 0.23 | 39.02 | 12.03 | 9.15 | 55.23 | 163.91 |
| Mal-MWENJE:201909 | 18.61 | 0.19 | 0.16 | 25.89 | 11.59 | 8.68 | 65.49 | 173.84 |
| Mwi-Baka:201909 | 18.41 | 0.22 | 0.18 | 31.49 | 11.52 | 8.67 | 60.23 | 183.8 |
| Mwi-CG 13:201909 | 22.98 | 0.18 | 0.13 | 27.27 | 10.37 | 8.38 | 56.9 | 178.4 |
| Mwi-Chitala:201909 | 19.90 | 0.16 | 0.12 | 20.74 | 10.87 | 8.48 | 62.05 | 179.49 |
| Mwi-CNG 1545:201909 | 18.90 | 0.21 | 0.16 | 28.18 | 11.64 | 9.80 | 56.83 | 142.95 |
| Mwi-ICG 14788:201909 | 19.12 | 0.18 | 0.14 | 21.96 | 11.54 | 9.73 | 64.77 | 160.51 |
| Mwi-ICG 5195:201909 | 20.22 | 0.25 | 0.20 | 35.18 | 10.29 | 7.14 | 58.69 | 183.15 |
| Mwi-ICG 6057:201909 | 19.70 | 0.17 | 0.14 | 20.03 | 13.90 | 12.02 | 52.84 | 188.79 |
| Mwi-ICG 6888:201909 | 21.86 | 0.15 | 0.12 | 24.04 | 10.99 | 8.65 | 62.59 | 189.15 |
| Mwi-ICGV-SM 03519:201909 | 17.93 | 0.30 | 0.25 | 40.13 | 12.22 | 10.74 | 58.03 | 162.2 |
| Mwi-ICGV-SM 05738:201909 | 18.03 | 0.31 | 0.26 | 36.69 | 10.47 | 8.21 | 55.86 | 138.58 |
| Mwi-ICGV-SM 08565:201909 | 17.90 | 0.19 | 0.16 | 25.08 | 11.92 | 8.80 | 59.36 | 177.25 |
| Mwi-ICGV SM 07504:201909 | 18.97 | 0.20 | 0.16 | 27.33 | 11.97 | 9.83 | 44.96 | 180.87 |
| Mwi-ICGV SM 07532:201909 | 17.37 | 0.29 | 0.26 | 36.97 | 13.66 | 10.78 | 47.96 | 127.8 |
| Mwi-ICGV SM 07533:201909 | 18.77 | 0.34 | 0.27 | 44.71 | 13.75 | 14.16 | 54.71 | 146.28 |
| Mwi-ICGV SM 07539:201909 | 17.43 | 0.32 | 0.28 | 40.35 | 10.77 | 8.91 | 54.18 | 157.66 |
| Mwi-ICGV SM 07544:201909 | 17.33 | 0.36 | 0.32 | 47.59 | 11.22 | 11.09 | 50.91 | 149.65 |
| Mwi-ICGV SM 08528:201909 | 18.67 | 0.38 | 0.30 | 46.69 | 11.23 | 9.43 | 59.75 | 168.07 |
| Mwi-ICGV SM 08533:201909 | 17.58 | 0.19 | 0.16 | 26.10 | 11.06 | 8.34 | 60.23 | 163.03 |
| Mwi-ICGV SM 09524:201909 | 20.81 | 0.18 | 0.14 | 25.79 | 12.64 | 10.57 | 55.69 | 182.67 |
| Mwi-ICGV SM 5521:201909 | 19.06 | 0.35 | 0.28 | 44.24 | 11.74 | 10.27 | 50.68 | 141.16 |
| Mwi-ICGV SM 99594:201909 | 19.44 | 0.29 | 0.24 | 47.99 | 11.11 | 8.89 | 49.93 | 167.93 |
| MZG-35B:201909 | 19.55 | 0.14 | 0.11 | 23.89 | 12.89 | 9.05 | 58.19 | 191.04 |
| MZG-75B:201909 | 19.37 | 0.25 | 0.21 | 33.31 | 10.25 | 6.93 | 52.53 | 179.26 |
| MZG-CBA 13001:201909 | 19.89 | 0.24 | 0.18 | 32.93 | 10.89 | 8.12 | 55.82 | 148.49 |
| MZG-ICG 12991:201909 | 19.23 | 0.21 | 0.18 | 30.00 | 12.56 | 8.70 | 52.86 | 179.9 |
| MZG-ICGV-SM 01513:201909 | 19.66 | 0.26 | 0.21 | 33.32 | 11.41 | 8.33 | 53.57 | 156.59 |
| MZG-ICGV-SM 03520:201909 | 19.49 | 0.19 | 0.15 | 18.50 | 12.53 | 10.01 | 62.98 | 172.17 |
| MZG-ICGV-SM 03530:201909 | 17.92 | 0.22 | 0.19 | 24.97 | 12.39 | 9.05 | 55.98 | 175.8 |
| MZG-ICGV-SM 08528:201909 | 21.24 | 0.15 | 0.12 | 20.19 | 11.36 | 8.98 | 62.42 | 177.01 |
| MZG-JL-24:201909 | 19.88 | 0.20 | 0.16 | 24.29 | 13.29 | 10.33 | 60.82 | 174.25 |
| MZG-NMP 13003:201909 | 18.56 | 0.28 | 0.23 | 36.08 | 9.06 | 6.79 | 58.99 | 176.44 |
| MZG-PAN-09001:201909 | 19.14 | 0.22 | 0.18 | 30.20 | 12.47 | 9.61 | 55.86 | 171.24 |
| MZG-PAN-09003:201909 | 18.73 | 0.12 | 0.10 | 15.72 | 12.40 | 8.56 | 59.72 | 185.45 |
| MZG-PAN-09021:201909 | 19.81 | 0.13 | 0.10 | 20.26 | 10.74 | 7.01 | 59.8 | 185.4 |
| MZG-PAN-09023:201909 | 18.50 | 0.14 | 0.12 | 17.47 | 11.24 | 7.89 | 62.64 | 192.75 |
| MZG-PAN-13004:201909 | 18.26 | 0.28 | 0.24 | 36.23 | 12.00 | 8.92 | 57.9 | 159.98 |
| MZG-PAN-13005:201909 | 17.38 | 0.27 | 0.24 | 35.72 | 11.99 | 8.60 | 54.27 | 163 |
| MZG-PAN-13006:201909 | 20.21 | 0.26 | 0.21 | 34.53 | 13.09 | 9.47 | 60.38 | 161.28 |
| MZG-PAN-13011:201909 | 18.66 | 0.21 | 0.17 | 27.24 | 11.60 | 9.00 | 62.53 | 172.8 |
| MZG-PAN-13014:201909 | 19.50 | 0.26 | 0.20 | 34.97 | 11.68 | 8.89 | 61.93 | 154.97 |
| Nig-0-20:201909 | 17.84 | 0.20 | 0.17 | 27.35 | 15.76 | 11.55 | 57.41 | 185.07 |
| Nig-55-437:201909 | 19.44 | 0.16 | 0.13 | 22.37 | 9.77 | 6.06 | 62.68 | 179.1 |
| Nig-796:201909 | 21.11 | 0.13 | 0.10 | 25.84 | 8.98 | 5.52 | 61.9 | 194.03 |
| Nig-ICGV-SM-99506:201909 | 19.32 | 0.16 | 0.13 | 21.24 | 9.27 | 6.35 | 60.86 | 172.5 |
| Nig-ICGV 8602:201909 | 17.87 | 0.24 | 0.20 | 32.65 | 9.68 | 6.37 | 60.26 | 170.66 |
| Nig-ICGV 87003:201909 | 18.42 | 0.21 | 0.18 | 30.06 | 11.19 | 8.27 | 59.69 | 172.56 |
| Nig-ICGV 91317:201909 | 19.18 | 0.26 | 0.21 | 40.28 | 12.06 | 8.20 | 58.63 | 168.9 |
| Nig-ICGV 91324:201909 | 19.49 | 0.17 | 0.14 | 25.63 | 12.21 | 9.57 | 65.8 | 185.63 |
| Nig-ICGV 94434:201909 | 18.47 | 0.17 | 0.14 | 23.58 | 10.92 | 8.53 | 60.93 | 180.22 |
| Nig-ICGVIS 07815:201909 | 18.42 | 0.25 | 0.20 | 33.16 | 12.39 | 9.82 | 52.95 | 175.65 |
| Nig-ICGVIS 07890:201909 | 18.64 | 0.24 | 0.20 | 33.74 | 11.40 | 9.12 | 51.82 | 182.72 |
| Nig-ICGVIS 07957:201909 | 20.78 | 0.22 | 0.18 | 34.11 | 12.64 | 11.33 | 57.94 | 173.17 |
| Nig-ICGVIS 07964:201909 | 19.02 | 0.27 | 0.22 | 34.91 | 10.18 | 7.67 | 58.57 | 164.17 |
| Nig-ICGVIS 07997:201909 | 18.87 | 0.16 | 0.13 | 22.04 | 12.11 | 10.26 | 52.83 | 178.57 |
| Nig-ICGVIS 07999:201909 | 19.99 | 0.28 | 0.23 | 39.91 | 12.55 | 9.93 | 58.21 | 180.18 |
| Nig-ICGVIS 79103:201909 | 19.21 | 0.20 | 0.16 | 29.51 | 13.01 | 10.64 | 53.06 | 172.07 |
| Nig-ICGVSM 99502:201909 | 20.55 | 0.20 | 0.16 | 25.63 | 11.83 | 10.75 | 58.69 | 173.04 |
| Nig-ICIAR 19-BT:201909 | 19.08 | 0.15 | 0.13 | 18.81 | 10.90 | 8.03 | 62.23 | 180.85 |
| Nig-NELSON-SPANISH:201909 | 20.41 | 0.23 | 0.19 | 31.39 | 10.46 | 7.93 | 57.11 | 174.68 |
| Nig-SRV1-3:201909 | 18.34 | 0.17 | 0.14 | 26.60 | 10.15 | 6.76 | 60.73 | 168.49 |
| Nig-T-DT1-2016:201909 | 19.06 | 0.20 | 0.17 | 26.45 | 9.73 | 7.18 | 55.25 | 183.91 |
| Nig-T-DT2-2016:201909 | 19.34 | 0.22 | 0.18 | 31.82 | 11.22 | 8.67 | 57.47 | 166.65 |
| Nig-T-EM1-2016:201909 | 21.22 | 0.13 | 0.10 | 19.84 | 13.21 | 9.60 | 59.2 | 170.27 |
| Nig-T-EM2-2016:201909 | 20.67 | 0.20 | 0.15 | 28.87 | 12.41 | 9.42 | 57.64 | 172.49 |
| Nig-T-RT1-2016:201909 | 24.03 | 0.14 | 0.10 | 19.26 | 9.33 | 6.38 | 56.5 | 186.26 |
| Nig-T1-95:201909 | 16.24 | 0.13 | 0.11 | 18.39 | 10.39 | 7.83 | 62.76 | 180.66 |
| Nig-T13-89:201909 | 19.53 | 0.23 | 0.18 | 32.21 | 11.13 | 7.63 | 56.95 | 174.21 |
| Nig-T14-89:201909 | 20.20 | 0.17 | 0.13 | 24.38 | 9.50 | 7.99 | 58.96 | 164.29 |
| Nig-T16-89:201909 | 18.76 | 0.14 | 0.11 | 18.28 | 10.18 | 6.99 | 57.98 | 191.42 |
| Nig-T169-83:201909 | 18.38 | 0.16 | 0.13 | 21.63 | 11.30 | 7.22 | 60.76 | 175.42 |
| Nig-T177-83:201909 | 18.32 | 0.10 | 0.08 | 18.73 | 9.86 | 7.25 | 58.36 | 195.13 |
| Nig-T183-83:201909 | 19.48 | 0.18 | 0.14 | 23.70 | 10.90 | 7.23 | 53.65 | 188.14 |
| Nig-T2-2007:201909 | 19.29 | 0.19 | 0.15 | 25.99 | 11.29 | 8.79 | 60.2 | 169.35 |
| Nig-T4-2006:201909 | 20.04 | 0.18 | 0.14 | 26.08 | 11.26 | 7.48 | 56.69 | 169.75 |
| Nig-T4-83:201909 | 27.25 | 0.13 | 0.09 | 23.23 | 11.75 | 7.78 | 62.76 | 178.92 |
| Nig-T4-89:201909 | 20.83 | 0.16 | 0.13 | 23.13 | 10.47 | 7.00 | 57 | 175.32 |
| Nig-T92-83:201909 | 21.34 | 0.16 | 0.12 | 22.71 | 10.28 | 6.94 | 63.96 | 175.22 |
| Nig-T95-83:201909 | 18.97 | 0.17 | 0.14 | 24.06 | 10.62 | 6.71 | 57.1 | 183.71 |
| Nig-TAIMAN-9:201909 | 20.23 | 0.22 | 0.17 | 30.31 | 12.13 | 9.25 | 56 | 174.38 |
| Nig-TM-H-94:201909 | 17.42 | 0.17 | 0.15 | 21.90 | 10.63 | 5.83 | 57.44 | 172.09 |
| Nig-TX903654:201909 | 17.89 | 0.17 | 0.14 | 24.69 | 9.29 | 6.08 | 56.69 | 189.53 |
| Nig-TX903796:201909 | 19.85 | 0.15 | 0.11 | 23.80 | 9.17 | 5.90 | 63 | 185.98 |
| Nig-TX903838:201909 | 20.53 | 0.13 | 0.10 | 24.68 | 10.44 | 6.91 | 64.34 | 203.67 |
| Oug-709 X VC74 RED UG:201909 | 18.71 | 0.16 | 0.13 | 14.55 | 11.57 | 8.74 | 59.29 | 184.27 |
| Oug-Acholi white:201909 | 18.18 | 0.19 | 0.16 | 22.55 | 10.16 | 6.73 | 47.9 | 165.06 |
| Oug-AWI 0802 RED UG:201909 | 18.85 | 0.27 | 0.21 | 35.40 | 11.09 | 8.64 | 56.37 | 167.61 |
| Oug-DOK 1 RED UG:201909 | 19.15 | 0.12 | 0.10 | 22.24 | 8.03 | 5.09 | 63.8 | 170.66 |
| Oug-ICGV SM 00537:201909 | 21.30 | 0.29 | 0.22 | 37.69 | 11.06 | 8.86 | 50.47 | 153.04 |
| Oug-ICGV SM 01504:201909 | 17.79 | 0.18 | 0.15 | 25.13 | 12.11 | 8.86 | 60.69 | 179.59 |
| Oug-ICGV SM 03590:201909 | 18.95 | 0.22 | 0.19 | 28.84 | 14.36 | 9.99 | 62.4 | 164.52 |
| Oug-ICGV SM 05650:201909 | 17.88 | 0.24 | 0.20 | 32.13 | 11.35 | 9.84 | 53 | 187.16 |
| Oug-ICGV SM 05702:201909 | 20.07 | 0.31 | 0.26 | 39.30 | 10.07 | 6.61 | 58.01 | 159.38 |
| Oug-ICGV SM 06518:201909 | 17.31 | 0.42 | 0.37 | 56.05 | 12.84 | 10.24 | 43.91 | 118.88 |
| Oug-ICGV SM 07540:201909 | 17.86 | 0.20 | 0.16 | 28.09 | 11.83 | 9.20 | 48.9 | 175.23 |
| Oug-ICGV SM 07593:201909 | 19.96 | 0.29 | 0.23 | 38.50 | 13.37 | 11.54 | 56.79 | 147.92 |
| Oug-ICGV SM 08556:201909 | 22.10 | 0.19 | 0.16 | 29.83 | 10.38 | 8.01 | 55.9 | 196.8 |
| Oug-ICGV SM 08572:201909 | 19.25 | 0.35 | 0.29 | 43.13 | 11.18 | 7.92 | 55.32 | 142.53 |
| Oug-ICGV SM 08577:201909 | 17.00 | 0.38 | 0.33 | 49.83 | 13.50 | 10.25 | 46.2 | 122.61 |
| Oug-ICGV SM 08586:201909 | 20.23 | 0.33 | 0.26 | 40.89 | 9.77 | 7.35 | 58.29 | 154.81 |
| Oug-ICGV SM 10005:201909 | 18.63 | 0.25 | 0.21 | 31.00 | 9.99 | 8.16 | 49.41 | 157.05 |
| Oug-ICGV SM 95360:201909 | 21.56 | 0.19 | 0.14 | 27.19 | 11.02 | 6.82 | 55.65 | 168.74 |
| Oug-ICGV SM 99537:201909 | 18.49 | 0.18 | 0.15 | 21.63 | 11.68 | 8.52 | 59.09 | 183.14 |
| Oug-ICGV SM 99551:201909 | 19.75 | 0.22 | 0.17 | 27.39 | 10.96 | 8.37 | 59.79 | 179.14 |
| Oug-ICGV SM 99555:201909 | 19.32 | 0.19 | 0.15 | 24.18 | 11.71 | 11.59 | 58.44 | 171.83 |
| Oug-KadonokhoX3590 Tan:201909 | 19.62 | 0.27 | 0.22 | 37.33 | 11.91 | 10.82 | 56.29 | 165.66 |
| Oug-RED BEAUTY UG:201909 | 19.65 | 0.12 | 0.10 | 19.95 | 11.88 | 10.74 | 62.33 | 181.12 |
| Oug-SGV 99046 UG:201909 | 22.14 | 0.35 | 0.26 | 45.17 | 12.50 | 8.28 | 58.15 | 161.03 |
| Oug-SGV ER 10010 UG:201909 | 17.27 | 0.23 | 0.20 | 29.17 | 10.29 | 6.95 | 54.73 | 143.36 |
| Sen-55-33:201909 | 21.16 | 0.16 | 0.13 | 22.82 | 10.41 | 6.62 | 56.35 | 185.17 |
| Sen-55-437:201909 | 20.29 | 0.16 | 0.13 | 21.63 | 10.02 | 6.42 | 60.94 | 188.83 |
| Sen-73-30:201909 | 21.91 | 0.22 | 0.16 | 30.64 | 10.77 | 7.23 | 59.59 | 151.77 |
| Sen-78-936:201909 | 18.24 | 0.12 | 0.10 | 22.12 | 9.32 | 6.36 | 62.71 | 187.63 |
| Sen-Boulkouss:201909 | 20.18 | 0.21 | 0.17 | 28.84 | 10.34 | 9.14 | 61.08 | 171.82 |
| Sen-DOGO_Chin2:201909 | 19.62 | 0.18 | 0.14 | 25.56 | 9.61 | 7.29 | 63.1 | 154.59 |
| Sen-DOGO_Chin6:201909 | 21.68 | 0.18 | 0.13 | 30.09 | 9.42 | 7.33 | 60.75 | 153.49 |
| Sen-DOGO_Chin8:201909 | 19.29 | 0.25 | 0.20 | 34.90 | 11.95 | 9.27 | 56.27 | 156.16 |
| Sen-Fleur 11:201909 | 22.68 | 0.25 | 0.19 | 35.27 | 11.61 | 8.84 | 62.51 | 178.75 |
| Sen-ICGV 96894:201909 | 17.56 | 0.30 | 0.27 | 50.19 | 16.76 | 13.13 | 51.34 | 131.55 |
| Sen-L4:201909 | 20.38 | 0.21 | 0.17 | 30.02 | 10.90 | 7.44 | 60.41 | 176.84 |
| Sen-LONGHU 1:201909 | 18.40 | 0.29 | 0.24 | 36.61 | 9.94 | 6.88 | 53.67 | 135.96 |
| Sen-Pungsan:201909 | 19.43 | 0.29 | 0.24 | 37.16 | 10.90 | 8.84 | 56.89 | 151.74 |
| Sen-Schubert:201909 | 18.99 | 0.24 | 0.20 | 30.76 | 11.64 | 8.27 | 56.52 | 173.29 |
| Sen-SERENUT 10R:201909 | 18.21 | 0.52 | 0.44 | 60.60 | 11.81 | 9.77 | 47.51 | 110.39 |
| Sen-Taaru:201909 | 18.28 | 0.18 | 0.15 | 26.43 | 8.99 | 6.10 | 60.96 | 176.67 |
| Tog-HG03:201909 | 19.61 | 0.23 | 0.18 | 23.79 | 12.62 | 9.43 | 60.4 | 147.85 |
| Tog-HG07:201909 | 18.96 | 0.12 | 0.09 | 14.81 | 13.37 | 9.44 | 60.38 | 189.57 |
| Tog-HG08:201909 | 23.85 | 0.23 | 0.15 | 31.40 | 12.50 | 9.54 | 60.45 | 160.01 |
| Tog-HG48:201909 | 18.24 | 0.24 | 0.19 | 31.46 | 11.89 | 9.62 | 55.78 | 165.37 |
| Tog-HG59:201909 | 18.67 | 0.12 | 0.10 | 19.65 | 11.10 | 8.23 | 60.4 | 192.08 |
| Tog-HG65:201909 | 19.38 | 0.34 | 0.28 | 43.89 | 11.63 | 10.01 | 53.24 | 151.54 |
| Tog-HG80:201909 | 19.39 | 0.22 | 0.18 | 27.64 | 11.34 | 9.11 | 49.34 | 166.17 |
| Tog-HG82:201909 | 20.36 | 0.23 | 0.18 | 32.61 | 11.00 | 8.04 | 58 | 165.96 |
| Tog-HG85:201909 | 20.55 | 0.20 | 0.16 | 27.89 | 11.27 | 7.55 | 57.06 | 170.84 |
| Tog-HG92:201909 | 17.28 | 0.21 | 0.18 | 27.27 | 11.79 | 9.78 | 61.98 | 160.07 |
| Tog-HG98:201909 | 19.06 | 0.20 | 0.17 | 28.64 | 11.50 | 8.83 | 56.86 | 183.44 |
| YENYAWOSO | 21.06 | 0.33 | 0.26 | 43.84 | 10.45 | 7.36 | 48.33 | 156.26 |
| Zam-12CS_121:201909 | 20.97 | 0.20 | 0.15 | 30.34 | 12.01 | 9.13 | 55.06 | 177.5 |
| Zam-6:201909 | 18.77 | 0.25 | 0.20 | 30.99 | 9.19 | 6.43 | 58.6 | 154.97 |
| Zam-8:201909 | 20.13 | 0.14 | 0.11 | 21.19 | 9.81 | 8.79 | 60.83 | 185.81 |
| Zam-ICGV-07541:201909 | 21.04 | 0.27 | 0.21 | 36.02 | 14.88 | 10.48 | 59.17 | 173.58 |
| Zam-ICGV-SM-06637:201909 | 19.35 | 0.35 | 0.28 | 47.88 | 10.99 | 9.62 | 53.32 | 178.31 |
| Zam-ICGV-SM-07517:201909 | 19.21 | 0.43 | 0.36 | 56.03 | 10.49 | 8.72 | 56.73 | 145.66 |
| Zam-KATETE:201909 | 19.79 | 0.17 | 0.14 | 25.83 | 9.86 | 6.49 | 60.93 | 189.41 |
| **MEAN** | **19.36** | **0.23** | **0.19** | **30.96** | **11.50** | **8.74** | **57.51** | **167.15** |
| **MIN** | **16.24** | **0.10** | **0.08** | **14.55** | **8.03** | **5.09** | **38.94** | **110.39** |
| **MAX** | **27.25** | **0.52** | **0.44** | **60.68** | **20.94** | **18.94** | **65.8** | **203.67** |
